# Health-Related Quality of Life Issues in Adolescents and Young Adults with Cancer: Discrepancies with the Perceptions of Health Care Professionals

**DOI:** 10.3390/jcm10091833

**Published:** 2021-04-22

**Authors:** Suzanne E. J. Kaal, Emma K. Lidington, Judith B. Prins, Rosemarie Jansen, Eveliene Manten-Horst, Petra Servaes, Winette T. A. van der Graaf, Olga Husson

**Affiliations:** 1 Department of Medical Oncology, Radboud University Medical Center, 6525 GA Nijmegen, The Netherlands; suzanne.kaal@radboudumc.nl (S.E.J.K.); Rosemarie.Jansen@radboudumc.nl (R.J.); 2 Dutch AYA ‘Young and Cancer’ Care Network, AYA IKNL, 3511 DT Utrecht, The Netherlands; e.manten-ayanationaal@iknl.nl (E.M.-H.); w.vd.graaf@nki.nl (W.T.A.v.d.G.); 3 Sarcoma Unit, Royal Marsden Hospital, London SW3 6JJ, UK; Emma.Lidington@rmh.nhs.uk; 4 Department of Medical Psychology, Radboud University Medical Center, 6525 GA Nijmegen, The Netherlands; judith.prins@radboudumc.nl (J.B.P.); petra.servaes@radboudumc.nl (P.S.); 5 Medical Oncology Department, Netherlands Cancer Institute—Antoni van Leeuwenhoek Hospital, 1066 CX Amsterdam, The Netherlands; 6 Department of Medical Oncology, Erasmus MC Cancer Institute, Erasmus University Medical Center, 3015 GD Rotterdam, The Netherlands; 7 Psychosocial Research and Epidemiology Department, Netherlands Cancer Institute—Antoni van Leeuwenhoek Hospital, 1066 CX Amsterdam, The Netherlands; 8 Institute of Cancer Research, London SM2 5NG, UK

**Keywords:** adolescent and young adult, AYA, cancer, health-related quality of life (HRQoL), health care professionals’ perception

## Abstract

This study aimed to identify health-related quality of life (HRQoL) issues of relevance for adolescent and young adult (AYA) cancer patients, the perception of relevant HRQoL issues for AYA in generalby the health care professionals (HCP), and discrepancies between issues identified by AYAs and HCP. Dutch AYAs aged 18–35 years at the time of cancer diagnosis (*N* = 83) and HCPs (*N* = 34) involved in AYA oncology were invited to complete the Quality of Life for Cancer Survivors questionnaire. HCPs rated physical symptoms such as fatigue, appetite, pain, constipation, sleep disorders, nausea and neuropathy among AYA cancer patients significantly higher (i.e., more problems) than AYA cancer patients. AYA cancer patients rated overall physical health and quality of life, happiness, satisfaction, usefulness and support from others, all positively formulated questions, significantly higher (i.e., more positive) than HCPs. The most highly rated issues (negative and positive combined) for AYAs were: perceived support from others, distress about initial cancer diagnosis, distress for family and overall quality of life. HCPs identified distress about diagnosis, treatment, family and interference of illness with employment/study as the most problematic issues (all negative) for AYAs. Five of the ten most common issues listed by patients and HCPs were the same. Male AYAs, patients treated with curative intent and those with a partner rated positive HRQoL issues higher than their counterparts. The discrepancy between the perception of patients and HCPs illustrates the importance of patient involvement in organizing physical and psychosocial care.

## 1. Introduction

Adolescents and young adults (AYA) diagnosed with cancer between the age of 18 and 35 are a distinct group compared to paediatric and adult oncology patients. Over the last few decades, the incidence of cancer in AYAs has increased in Europe as well as in the United States and Canada [1,2,3]. In the Netherlands, about 2700 new patients per year are diagnosed with cancer in this age group, which is 3% of all invasive cancer diagnoses [4]. While improvements in cancer survival in AYA patients lag behind those in children and adults, about 80% of AYA cancer patients in the Netherlands will survive at least five years [5].

These AYA survivors are in a phase of life where they normally reach developmental milestones such as completing education, establishing intimate relationships, pursuing gainful employment and having children [6]. Cancer poses a major challenge to achieving these milestones. Problems concerning self-esteem, autonomy, body image, fertility and sexuality may have a negative impact on health-related quality of life (HRQoL) in AYA cancer patients [6,7,8]. Two recent review papers show that AYA with cancer experience worse HRQoL compared to the general population [9,10]. Risk factors for poor HRQoL in AYAs include low socio-economic status, tumour type (patients diagnosed with tumor with poor prognosis report worse HRQoL), treatment status, female sex, unemployment, high levels of distress and physical symptoms [11,12]. Measuring HRQoL in AYAs allows physicians to better manage the complexity of caring for AYAs with cancer [10,13,14,15].

In recent years, there has been increasing interest in patient-reported outcomes (PROs), including HRQoL, in AYA cancer patients [10]. A PRO is defined as information about the status of a patient’s health condition that comes directly from the patient, without interpretation of the patient’s response by a clinician or anyone else [16]. PROs are generally measured using structured questionnaires that evaluate a number of related domains using scales comprised of multiple items which are validated in large populations and psychometrically tested [17]. HRQoL includes multiple domains such as physical, psychological, social, and spiritual well-being [18,19]. While health researchers analyse and report HRQoL in these domains as this is the level psychometrically validated, it can lead to loss of information about the specific issues faced by patients [20,21]. Evaluating HRQoL on item level provides detail about which issues result in high or low domain scores and can help identify specific areas of need.

To achieve optimal patient-centred care, it is essential that health care professionals (HCPs; medical and allied health care professionals) understand AYAs’ needs and preferences. A previous study in Australia showed that most oncology professionals recognized the unique needs of AYAs (defined as 15–25 years old) with regard to biological, genetic, epidemiological, psychological, social and cultural factors [22]. However, HCPs broadly misconstrued the priorities of AYAs. HCPs believed AYAs would rank survival outcomes, concerns about death and dying, and functional well-being as the most salient issues following a cancer diagnosis. However, AYAs actually prioritised issues associated with normal life stage development including peer and family relationships, engagement in education and employment, development of autonomy and treatment environment (e.g., in- or outpatient, facilities and staff) [22]. This study showed significant discrepancies between patient priorities and HCP perceptions in younger AYAs. However, it is unclear if discrepancies exist in the older AYA cohort up to age 35. One could argue that this age group has reached another life phase with different milestones, more responsibilities and more independence from parents in comparison to AYAs aged 15–25 years [7] which may be more or less aligned with the understanding of HCPs. In addition, it is important to investigate whether this incongruence exists across health care systems, such as the Dutch system, where there is a clear distinction between paediatric and adult oncology.

Identifying HRQoL priorities and providing care to meet these needs may lead to higher patient satisfaction and can improve doctor-patient relationships and ultimately overall quality of care [23]. Therefore, the aims of this exploratory study are to identify: (1) the ten most common health-related quality of life (HRQoL) issues in adolescent and young adult (AYA) cancer patients (according to sex, cancer type, treatment intention, partner and family status); (2) the ten most common HRQoL issues of AYAs according to the perceptions of health care professionals (HCP); (3) discrepancies between HRQoL issues identified by AYAs and HCP.

## 2. Materials and Methods

### 2.1. Participants

Patients were invited to participate if they were aged 18 to 35 years old at the time of cancer diagnosis and had been seen by at least one member of the AYA team at the Radboud university medical center (Radboudumc) in the Netherlands. The Radboudumc is an expert center in delivering age-specific care for AYA cancer patients during and after cancer treatment. The AYA team is a dedicated multidisciplinary team including a medical oncologist, clinical nurse specialist, medical psychologist, social worker and clinical occupational health physician. Patients receive standard medical care from their own treating physician in the Radboudumc (e.g., medical oncologist, haematologist, etc.) and visit the AYA team for age-specific questions and care needs. In general, patients attending the AYA service have high disease severity, advanced disease, intensive treatments, and high psychological needs. Eligibility for the study did not include treatment status (e.g., on or off treatment), type of treatment (e.g., surgery, chemotherapy, etc.), or the number of AYA team visits to reflect the heterogeneous population of AYA cancer patients visiting the AYA team. Inclusion commenced January 2012 and ended March 2016.

HCPs were eligible to participate if they were involved in the care of AYA cancer patients in the Netherlands. HCPs were invited to participate in person at the 2017 annual AYA congress on age-specific care. In addition, HCP affiliated with the Dutch AYA ‘Young and Cancer’ Care Network (www.ayazorgnetwerk.nl (accessed on 20 March 2021)) were invited to participate via email.

### 2.2. Procedure

Potential patient participants were recruited by postal invitation. Patients willing to participate responded to a member of the AYA team by email and provided informed consent. The AYA team member provided a link to all participants by e-mail to complete the questionnaire online. No reminders were sent. The research ethics committee determined the study did not require full review and approval (CMO Regio Arnhem-Nijmegen, #2016-2872).

### 2.3. Measures

#### 2.3.1. Sociodemographic and Clinical Characteristics

Demographic data, including age, sex, partnership, number of children, living situation, educational attainment and employment status were self-reported by the patient. Medical data, including tumour type, disease stage, treatments, treatment status at participation (on/off treatment), treatment intent, and time since initial diagnosis were extracted from medical records by one of the researchers (SK).

HCPs also self-reported background information including age, gender, profession, hospital and the number of AYA cancer patients seen per month.

#### 2.3.2. Health-Related Quality of Life

##### AYA Cancer Patients

Patient participants completed the modified version of the Quality of Life for Cancer Survivors (QoL-CS) questionnaire [24] which originally consists of 41 items measuring the negative and positive impact of cancer in four domains: physical, mental, social and spiritual. Respondents answer each item on an interval rating scale ranging from 0 to 10. We adapted the questionnaire by adding four items regarding neuropathy (a burning or tingling sensation or a feeling of numbness), concerns about dying, concerns about fertility and concerns that family members will get cancer. These were added to reflect issues that were faced in daily clinical practice and frequently reported in our multidisciplinary AYA team meetings. The items regarding fear of recurrence and spreading (metastases) were combined into 1 item to make the question applicable for everyone to answer. Therefore, the total number of items is 44. Questions focus on negative impacts, with higher scores representing worse scores, or positive impacts, with higher scores representing better scores. An example of a negative question in the physical domain: ‘To what extent are sleep changes a problem for you?’ (0 = no problem, 10 = severe problem). An example of a positive impact question: ‘How useful do you feel?’ (0 = not at all, 10 = extremely). Three items (importance of religious activities, importance of spiritual activities and spiritual change) were excluded from our analyses, as they reflect religious aspects and their intrinsic value differs per person dependent on cultural and religious background.

HCPs were asked to complete the modified QoL-CS questionnaire according to the extent they believed AYA patients experienced each issue.

## 3. Statistical Analysis

Analyses were performed using SPSS statistical software (version 24, Chicago, IL, USA) and two-sided *p* values of <0.05 were considered statistically significant. Differences in HRQoL items between two groups were compared with an independent t-test and between more than two groups with an analysis of variance (ANOVA). For AYA cancer patients, HRQoL items were analysed according to five variables: age (emerging adult (18–25 years)/young adult (26–35 years at diagnosis), gender (male/female), cancer type (testicular, sarcoma, breast, hematologic, gynaecologic and other), treatment intent (curative/palliative), disease phase (undergoing active treatment/completed treatment), partner (yes/no) and children (yes/no). Clinical significance was determined by applying Normans rule of thumb (observed difference exceeds half mean standard deviation) [25].

## 4. Results

### 4.1. Patient Sociodemographic and Clinical Characteristics

A total of 89 AYAs completed the online questionnaire. This amounted to 57% of those who responded to the letter invitation (155 patients) and 29% of the total AYAs invited (309 patients). A small proportion of patients did not participate due to technical problems. Six patients were excluded due to ineligibility based on age: four were younger than 18 years when diagnosed with cancer and two were older than 35 years at the time of diagnosis. Table 1 displays the sociodemographic, disease, and treatment-related characteristics of the final sample (83 patients). The mean age at the time of diagnosis was 27.5 (SD = 4.6) years. The average time since diagnosis was 2.1 years (SD = 2.6), 71 (86%) patients received curative treatment and 43 (52%) were male. The common diagnoses were testicular cancer (34%) and sarcoma (19%).

### 4.2. Health Care Professionals Characteristics

Thirty-four HCPs from 12 hospitals completed the QoL-CS questionnaire. Respondents included 11 medical oncologists, 11 nurses, five nurse specialists, two psychologists, one social worker, one physical therapist, one rehabilitation specialist, and one who did not report his/her medical profession. The mean age of the HCPs was 45 years (range 23–64 years) and four (12%) were male. The HCPs saw an average of 15 (range 1–70) AYA cancer patients per month. Four HCPs were from non-university hospitals.

### 4.3. HRQoL Items

Table 2 shows the mean score on each HRQoL item rated by AYA cancer patients and HCPs. HCPs rated physical symptoms like fatigue (7.3 vs. 2.7; *p* < 0.001), appetite (5.1 vs. 1.5; *p* < 0.001), pain (4.3 vs. 2.3; *p* < 0.001), constipation (3.7 vs. 1.8; *p* < 0.001), sleep disorders (5.2 vs. 2.8; *p* < 0.001), nausea (4.8 vs. 1.6; *p* < 0.001) and neuropathy (4.8 vs. 2.2; *p* < 0.001) among AYA cancer patients significantly higher than AYA cancer patients. The differences for each of these items were clinically relevant. AYA cancer patients rated overall physical health (6.6 vs. 5.7; *p* = 0.004) and quality of life (7.2 vs. 6.2; *p* = 0.003), happiness (7.1 vs. 6.2; *p* = 0.012), satisfaction (6.8 vs. 5.9; *p* = 0.012), usefulness (6.4 vs. 5.3; *p* = 0.014) and support from others (8.1 vs. 6.2; *p* < 0.001), all positively formulated questions, significantly higher than HCPs. HCPs rated distress about time since treatment completion (6.9 vs. 5.5; *p* = 0.014), anxiety (6.5 vs. 3.7; *p* < 0.001), depression (5.0 vs. 3.0; *p* < 0.001), fear of future tests (6.9 vs. 4.7; *p* < 0.001), fear of recurrent cancer (7.1 vs. 5.4; *p* = 0.001), fear of dying (6.2 vs. 4.1; *p* = 0.001), problems with personal relationships (6.6 vs. 3.7; *p* < 0.001), sexuality (7.2 vs. 4.3; *p* < 0.001), concerns about fertility (7.3 vs. 5.0; *p* = 0.001), interference with illness of employment/study (7.8 vs. 6.2; *p* = 0.004), feeling isolated (6.1 vs. 3.7; *p* < 0.001), uncertainty about the future (6.6 vs. 5.2; *p* = 0.010) and life purpose (6.1 vs. 4.5; *p* = 0.005) as clinically and statistically significantly more problematic HRQoL items than AYAs.

Table 3a shows the top ten most highly rated HRQoL issues by AYA cancer patients and HCPs and the overlapping items in the middle column. Support from others appeared as the most highly scores HRQoL item among AYA cancer patients, followed by distress about initial cancer diagnosis and distress for family. These last two items were scored as most important by HCP. Only five out of ten items were similar for patients and HCP. Six out of ten items of AYA cancer patients were positively formulated. Table 3b displays top ten most highly rated HRQoL items by AYA cancer patients and HCP for only negatively formulated items. It shows that the first three items are similar for both groups (distress about cancer diagnosis, distress for family and distress about cancer treatment). Six out of ten items overlapped.

Table 4a–c depict the top ten most commonly experienced HRQoL items for AYA cancer patients according to gender, cancer type, treatment intention, partner, having children, disease phase and age group. It shows that male AYA cancer patients scored significantly higher on items concerning quality of life (*p* = 0.019), happiness (*p* = 0.004), satisfaction (*p* = 0.025), hopefulness (*p* = 0.009), overall physical health (*p* = 0.002) and experienced less interference with activities at home (*p* = 0.022). Between tumour types there were differences on the items: quality of life (*p* = 0.023), happiness (*p* = 0.012), cancer treatment distress (*p* = 0.027), overall physical health (*p* = 0.004) and interference with activities at home (*p* = 0.020). For sarcoma patients these effects were larger than for patients with other cancer types. AYA cancer patients treated with curative intention scored significantly higher on items concerning quality of life (*p* = 0.006), hopefulness (*p* = 0.000) and overall physical health (*p* = 0.000) in comparison with patients treated with palliative intent. AYA patients with a partner scored significantly higher on happiness (*p* = 0.008), satisfaction (*p* = 0.013) and overall physical health (*p* = 0.049). The difference in overall physical health between groups was not clinically relevant. There were no differences in top ten most experienced items when stratified for having children or not, disease phase (active vs. completed treatment) and age group (emerging adults vs. young adults).

## 5. Discussion

The results of the current study indicate that there is a considerable discrepancy in the level of impact cancer and its treatment have on HRQoL in AYA cancer patients and the level perceived by HCPs. AYA cancer patients rated most HRQoL items as less problematic than HCPs, indicating that professionals believe that the burden of cancer and treatment is larger than it is actually perceived by patients themselves. This holds for all four domains (physical, psychological, social and spiritual) of the QoL-CS questionnaire. Top ten items for AYA cancer patients are dominated by six positively formulated questions, indicating that AYA cancer patients are more inclined to emphasize HRQoL items with a positive connotation like happiness, satisfaction, hopefulness and social support. In our study, stratified analyses showed that male patients, patients being treated with curative intent and patients who have a partner valued positive formulated HRQoL items higher.

Our results are in line with previous studies. Thompson et al. found that there is a significant gap between the identified health care preferences of AYA with cancer (15–25 years) and the understanding of the Australian oncology HCP who deliver their care. HCP significantly underestimated the breadth of psychosocial concerns in AYA with cancer and believed they focused on survival and physical wellbeing [22]. In contrast, a study among older patients (mean age 55 years) found there was considerable consistency between physicians’ and patients’ perceptions of the needs and support that the patients received [23]. There was, however, a discrepancy between the actual and desired level of emotional and cognitive support. Another study found that the issues older cancer patients (mean age 61.5 years) preferred to be addressed, were the issues that HCP felt difficult to deal with [26].

A study among 294 long-term breast cancer survivors almost 6 years after cancer diagnosis (mean age 50.9 years) using the QoL-CS questionnaire showed that survivors experienced a negative impact on overall quality of life but also benefits including hopefulness, having a life purpose and having a positive change after treatment, which helped to cope with poorer outcomes [27]. However, in our study HCP scored life purpose and positive change higher than AYA. In our study we also observed many of these positive effects, therefore it could be hypothesized that resilience and post-traumatic growth can be important consequences in the AYA population after cancer treatment [28,29].

Male AYA cancer patients scored higher on items concerning quality of life, happiness, hopefulness, overall physical health and experienced less interference with activities at home to a clinically relevant level. This is in line with a previous study where male gender showed a positive correlation with empowerment, which may be related to the use of different coping strategies [30]. It could also be due to the fact that a substantial part of our study sample includes male patients with testicular cancer (34%) who, in general, have a good prognosis and a relatively short treatment duration. AYA patients with partners scored higher on happiness and satisfaction to a clinically relevant level, which illustrates the buffering and stabilizing effect of the social support of a partner [31]. Interestingly, no differences were found for disease phase (active v. completed treatment) and age group (emerging adult vs. young adult). It could be that these differences are not expressed in the top 10 HRQoL items, but in the other items that we have not been examined in detail. This is supported by the findings of a recent study were no significant differences in any HRQoL domain by cancer type, and few significant differences were observed in PROMIS domains between developmental groups among on-treatment AYA survivors. In contrast, off-treatment emerging adults and young adults reported significantly higher symptoms and worse functioning compared to adolescents [32].

It is valuable to identify the HRQoL areas where AYA cancer patients experience the most problems. The discrepancy between the level HRQoL problems reported between AYAs and HCPs illustrates that we must invest in clarifying patient needs and in training to deliver age-specific psychosocial care for oncology HCPs. The results of this study will add to the growing body of work in the needs of AYA oncology patients and support the tailoring of AYA-specific care. It also emphasizes the importance of patient experiences and needs in the development of age-specific health care services [33].

Since 2013, the Dutch AYA ‘Young and Cancer’ Care Network has been established through co-creation with AYA cancer patients and HCPs from university medical centres and large regional centres. The main goal of this AYA Care Network is to improve the care for and the quality of life of AYA cancer patients by developing structural, standardized, comprehensive and patient-centred guidelines for AYA cancer care, research and education. An e-module has been developed recently to educate students and health care professionals in the Netherlands on age-specific topics relevant to AYA cancer patients. Better training of HCPs can reduce discrepancies in perceptions of AYA supportive care needs between patients and HCPs.

We acknowledge several limitations of our study. First, AYA cancer patients responded based on their individual experience, while HCPs evaluated the HRQoL of the AYA cancer patient population in general not referring to a specific time after diagnosis, disease or treatment stage. HCPs may have responded based on patients currently undergoing treatment, while most of the patients were around two years from diagnosis. Furthermore, the HCP sample was composed primarily with professionals from the medical oncology discipline, with limited inclusion of allied health professionals including psychosocial providers. Both, may also have skewed the focus on symptoms among the HCPs and may under-represent more psychosocial aspects of an AYAs’ HRQoL. Second, AYA cancer patients were treated in a single centre and received multidisciplinary care by a dedicated AYA team, while the HCPs were from several centres. It could be that HRQoL item scores are better than those of AYA cancer patients treated in other centres in the Netherlands without age-specific care. The patients in the current study sample were diagnosed with a relatively advanced stage of disease and were treated intensively, mostly with more than one treatment modality. This is an overestimation in disease severity of the entire AYA cancer population [34]. Both factors limit the generalizability of the results of the current study.

Third, the QoL-CS questionnaire is not an AYA-specific HRQoL questionnaire. It was validated in a sample of cancer survivors with a mean age of 49.6 years who were on average 6.7 years after cancer diagnosis [24]. Therefore, we assume that the QoL-CS questionnaire has less discriminative value in our AYA population as our sample was a median of 2.1 years after cancer diagnosis. An AYA-specific HRQoL questionnaire was not in place when we started with this research. In the meantime, a few AYA specific questionnaires have been developed. For example, adult and paediatric measures have been adapted to the specific issues relevant to AYAs and have AYA versions (PedsQL) [35] and there are measures which have been developed with and designed specifically for AYAs (CNQ-YP) [36]. In response to the need for AYA specific questionnaires the EORTC Quality of Life Group are also developing the EORTC-QLQ-AYA module with AYA specific issues (e.g., impact on family, dependency on others, interrupted education, workability) [37]. The first phase of the questionnaire development showed that AYAs with cancer have to deal with disrupted life plans and difficulty establishing romantic relationships which are likely to be more common to AYAs with cancer and might not be captured by the tool used in this study [37]. The Impact Of Cancer AYA (IOC-AYA) questionnaire has shown good psychometric properties to measure positive and negative HRQoL aspects in the AYA population [14] and could, therefore, also be an asset in future research using AYA-specific questionnaires. Moreover, studies have shown that the meaning of patients’ self-evaluations of their quality of life may not be the same across different points in time, a phenomenon known as response shift. It reflects change that occurs because of adaptation to cancer, not true change due to cancer progression [38]. This phenomenon could have influenced our results.

A fourth limitation is the low response rate and potential sampling bias, which is not unusual in studies in young patients with cancer. Our study, however, had an even lower response than previous questionnaire studies among AYAs (29% response rate in the current study sample versus 43% and 52% in previous studies) [39,40]. Unfortunately, we do not have information regarding the reasons for not participating. As demographic and clinical data were not collected from the non-responders, we could not rule out selection bias. The results of our exploratory study should be interpreted with caution, the low number of participants limited us to perform multivariate analyses and apply Bonferroni corrections.

A fifth limitation is that we have used the QoL-CS questionnaire in a group of oncology HCPs. As the QoL-CS questionnaire was validated in cancer survivors [24], one could question whether the HCPs interpreted the questions in a similar way. Additionally, HCPs may not have been able to estimate the effect on HRQoL for AYAs across cancer types as they may only have treated a few patients with specific types of cancer. Sixth, the QoL-CS is a cancer-generic HRQoL questionnaire. We might have missed some AYA-specific and/or disease-specific HRQoL issues. There is an ongoing debate in the literature on the optimal HRQoL measurement strategy for AYAs given their heterogeneity in terms of cancer types and developmental stages [41,42]. A core item set combined with the advantages of a flexible measurement system will support future collaborative and comparative research efforts to improve AYA health outcomes.

Seventh, the HCP sample was relatively small and only included a few physicians. As most of the data were gathered at an AYA conference, one could assume that HCPs in the sample were highly dedicated and informed in the care of AYA cancer patients, which could skew the data.

In conclusion, this exploratory study showed incongruence between the views of AYA cancer patients and HCPs regarding the most problematic HRQoL items. Overall, HCPs in this study believed that the burden of cancer and its treatment is bigger than actually perceived by the patients themselves. Assessing HRQoL can, therefore, be of clinical importance in order to provide optimal age-specific care. The discrepancy between patients and HCP illustrates the importance of co-creation of physical and psychosocial survivorship care for this age group. Future research should use an age-specific questionnaire, which discriminates between positive and negative HRQoL items and its impact on overall HRQoL in AYA cancer patients.

## Figures and Tables

**Table 1:** Demographic and clinical data of AYA cancer patients.

	AYA Cancer Patients *N* = 83
Gender	
Male	43 (52%)
Female	40 (48%)
Age at diagnosis, mean	27.5 (4.6)
Age at diagnosis	
Emerging adult (18–25 years)	30 (36%)
Young adult (26–35 years)	53 (64%)
Age at survey	29.6 (4.8)
Time since cancer diagnosis	2.1 (2.6)
Cancer Diagnosis	
Testicular cancer	28 (34%)
Sarcoma	16 (19%)
Breast cancer	10 (12%)
Lymphoma/leukaemia	10 (12%)
Gynaecological cancer	9 (11%)
Melanoma	3 (4%)
Other *	7 (8%)
Stage	
NA	9 (11%)
Stage 1	11 (13%)
Stage 2	25 (30%)
Stage 3	13 (16%)
Stage 4	18 (22%)
Unknown	7 (8%)
Treatment Intention	
Curative	71 (86%)
Palliative	12 (14%)
Treatment Status at Participation	
Active	22 (27%)
Completed	61 (73%)
Treatment Type	
Surgery	70 (84%)
Chemotherapy	72 (87%)
Radiotherapy	24 (29%)
Immunotherapy/targeted therapy	13 (16%)
Hormonal therapy	7 (8%)
Systemic therapy other	13 (16%)
Partner	
Yes	58 (70%)
No	24 (29%)
Children	
Yes	27 (33%)
No	55 (66%)
Living Situation	
With parents	14 (17%)
On own	24 (29%)
With partner	44 (53%)
Highest Completed Education	
Low/intermediate vocational education or less	38 (46%)
High-level vocational education and/or university	44 (53%)
Employed/Studying	
Yes	68 (82%)
No	15 (18%)

Not all numbers add up to 83 because of missing data * Other cancer types comprise brain tumour (*n* = 1), sigmoid carcinoma (*n* = 1), oropharyngeal cancer (*n* = 1), neuroendocrine tumour (*n* = 1), salivary gland cancer (*n* = 1), adrenal carcinoma (*n* = 1), lung cancer (*n* = 1).

**Table 2:** Mean scores (SD) on HRQoL items of AYA cancer patients and health care professionals (scale range 0–10).

	AYA (*n* = 83) Mean (Standard Deviation)	Health Care Professionals (*n* = 34) Mean (Standard Deviation)	*p*-Value
Physical Well-Being			
Fatigue	4.4 (2.7)	7.3 (1.2)	0.000 **^a^
Appetite	1.5 (2.2)	5.1 (2.3)	0.000 **^a^
Pain	2.3 (2.3)	4.3 (2.0)	0.000 **^a^
Constipation	1.8 (2.4)	3.7 (1.8)	0.000 **^a^
Sleep	2.8 (2.8)	5.2 (1.9)	0.000 **^a^
Nausea	1.6 (2.6)	4.8 (2.3)	0.000 **^a^
Menstrual changes	3.7 (3.8)	5.2 (2.5)	0.074
Neuropathy	2.2 (2.5)	4.8 (2.6)	0.000 **^a^
Overall physical health ^b^	6.6 (1.6)	5.7 (1.4)	0.004 **^a^
Psychological Well-Being			
Coping difficulties	4.1 (2.6)	6.2 (1.5)	0.000 **^a^
Quality of life ^b^	7.2 (1.7)	6.2 (1.1)	0.003 **^a^
Happiness ^b^	7.1 (1.9)	6.2 (1.3)	0.012 *^a^
Control ^b^	5.5 (2.6)	5.1 (1.6)	0.411
Satisfaction ^b^	6.8 (1.9)	5.9 (1.3)	0.012 *^a^
Concentration/memory ^b^	5.7 (2.2)	5.0 (1.6)	0.095
Usefulness ^b^	6.4 (2.3)	5.3 (1.6)	0.014 *
Appearance	5.0 (3.0)	6.2 (2.0)	0.030 *
Self-concept	3.8 (2.8)	6.4 (1.7)	0.000
Distress about initial diagnosis	7.6 (2.5)	8.5 (1.5)	0.054
Cancer treatment distress	6.9 (2.3)	7.8 (1.5)	0.047 *
Distress about time since treatment completion	5.5 (2.8)	6.9 (1.4)	0.014 *^a^
Anxiety	3.7 (2.7)	6.5 (1.3)	0.000 **^a^
Depression	3.0 (2.6)	5.0 (1.3)	0.000 **^a^
Fear of future tests	4.7 (2.7)	6.9 (1.4)	0.000 **^a^
Fear of second cancer	4.8 (2.9)	5.7 (2.1)	0.107
Fear recurrent cancer	5.4 (2.5)	7.1 (1.2)	0.001 **^a^
Fear of dying	4.1 (3.2)	6.2 (1.8)	0.001 **^a^
Social Well-Being			
Family distress	7.6 (2.0)	7.8 (1.1)	0.671
Support from others ^b^	8.1 (1.8)	6.2 (1.6)	0.000 **^a^
Personal relationships	3.7 (2.9)	6.6 (1.6)	0.000 **^a^
Sexuality	4.3 (3.3)	7.2 (1.2)	0.000 **^a^
Concerns about fertility	5.0 (3.6)	7.3 (1.8)	0.001 **^a^
Interference of illness with employment or study	6.2 (3.0)	7.8 (1.2)	0.004 **^a^
Home activities	6.5 (2.7)	7.2 (1.2)	0.105
Feel isolated	3.7 (3.0)	6.1 (1.5)	0.000 **^a^
Financial burden	6.3 (3.0)	5.9 (1.6)	0.533
Concerns that family members will get cancer	3.9 (2.8)	4.8 (1.7)	0.097
Spiritual Well-Being			
Importance of religious activities ^c^	2.1 (3.3)	3.6 (1.9)	0.020 *^a^
Importance of spiritual activities ^c^	1.3 (2.2)	4.4 (2.0)	0.000 **^a^
Spiritual change ^c^	1.8 (2.4)	4.9 (1.8)	0.000 **^a^
Uncertainty future	5.2 (2.9)	6.6 (1.6)	0.010 *^a^
Illness positively changed life ^b^	5.1 (2.8)	6.2 (1.6)	0.028 *
Life purpose ^b^	4.5 (3.1)	6.1 (1.6)	0.005 **^a^
Hopefulness ^b^	6.8 (2.2)	7.3 (1.2)	0.169

* *p* < 0.05; ** *p* < 0.01; ^a^ = clinical relevant; ^b^ = Positively formulated item; All other items are negatively formulated: the higher the score, the bigger the problem; ^c^ = neutral formulated items because of religious background, not in analysis.

**Table 3:**
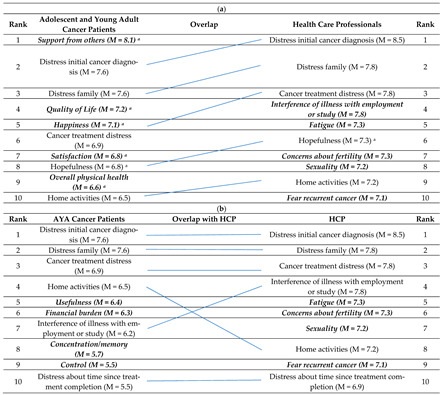
(**a**) Top 10 most important HRQoL issues among AYA cancer patients and health care professionals including positive formulated items. (**b**) Top 10 most important HRQoL issues among AYA cancer patients and health care professionals excluding positive formulated items.

(**a**) Unique items for AYA cancer patients or HCP are in ***bold and italics***; ^a^ = positively formulated questions; M = mean of the item; AYA = adolescent and young adult; HCP = health care professional. (**b**) Unique items for AYA cancer patients or for HCP are in ***bold and italics***; M = mean of the item; AYA = adolescent and young adult; HCP = health care professional.

**Table 4:** (**a**) Most important HRQoL items (mean (SD)) among AYA cancer patients (*n* = 83) for gender and cancer type. (**b**) Most important HRQoL items (mean (SD)) among AYA cancer patients (*n* = 83) for treatment intention, partner and children. (**c**) Most important HRQoL items (mean (SD)) among AYA cancer patients (*n* = 83) for disease phase and age.

**(a)**										
	**Gender**			**Cancer Type**						
	**Male**	**Female**	***p*-Value**	**Testicular**	**Sarcoma**	**Breast**	**Hematologic**	**Gynaecologic**	**Other**	***p*-Value**
Support from Others	8.4 (1.6)	7.8 (1.9)	0.082	8.6 (1.6)	8.1 (1.7)	7.2 (1.9)	8.1 (2.7)	7.5 (1.3)	8.1 (1.5)	0.351
Distress Initial Cancer Diagnosis	7.4 (2.3)	7.7 (2.6)	0.635	7.5 (2.2)	6.9 (2.6)	7.7 (3.3)	6.1 (3.0)	8.1 (1.7)	8.7 (1.9)	0.148
Distress for Family	7.7 (1.9)	7.5 (2.1)	0.62	7.5 (1.8)	8.4 (1.5)	7.0 (2.9)	6.7 (2.5)	7.3 (2.0)	8.5 (1.3)	0.153
Quality of Life	7.6 (1.5)	6.7 (1.8)	0.019 *^a^	8.0 (1.4)	6.8 (1.8)	7.2 (1.0)	7.2 (1.2)	6.3 (2.3)	6.4 (2.1)	0.023 * ^b,c,d,e,f,g,^
Happiness	7.6 (1.6)	6.5 (2.0)	0.004 **^a^	7.8 (1.8)	7.2 (1.4)	7.4 (1.4)	7.3 (1.3)	5.9 (2.2)	5.9 (2.2)	0.012 * ^c,d,e,f,g,h,i,j^
Cancer Treatment Distress	6.7 (2.0)	7.1 (2.6)	0.428	6.6 (2.0)	7.5 (2.1)	5.9 (3.3)	5.6 (2.6)	8.3 (1.2)	7.9 (1.8)	0.027 * ^c,d,f,g,i,j,k,l^
Satisfaction	7.3 (1.8)	6.4 (2.0)	0.025 *	7.5 (1.9)	6.5 (2.0)	6.9 (1.4)	7.4 (1.6)	6.0 (2.3)	6.1 (2.0)	0.14
Hopefulness	7.4 (1.9)	6.1 (2.4)	0.009 **^a^	7.7 (1.6)	5.9 (2.6)	7.2 (1.5)	6.5 (2.0)	5.9 (2.7)	6.1 (2.6)	0.063
Overall Physical Health	7.1 (1.4)	6.0 (1.6)	0.002 **^a^	7.5 (1.3)	5.9 (1.2)	6.6 (1.2)	6.5 (1.4)	6.1 (1.7)	5.8 (1.8)	0.004 ** ^b,c,d,i,m,n^
Interference Activities at Home	5.8 (2.8)	7.2 (2.5)	0.022 *^a^	5.1 (2.7)	8.2 (1.7)	6.9 (1.7)	6.3 (3.1)	6.5 (3.2)	7.4 (2.4)	0.020 * ^b,c,d,e,k,l,m^
**(b)**										
	**Treatment Intention**			**Partner**			**Children**			
	**Curative**	**Palliative**	***p*-Value**	**Yes**	**No**	***p*-Value**	**Yes**	**No**	***p*-Value**	
Support from Others	8.1 (1.8)	8.3 (1.1)	0.745	8.4 (1.5)	7.6 (1.6)	0.038	7.8 (1.8)	8.4 (1.4)	0.102	
Distress Initial Cancer Diagnosis	7.4 (2.6)	8.8 (1.3)	0.071	7.9 (2.3)	7.1 (2.4)	0.166	8.2 (2.1)	7.4 (2.4)	0.122	
Distress for Family	7.5 (2.1)	8.6 (1.3)	0.076	7.7 (2.0)	7.5 (2.0)	0.764	7.7 (1.7)	7.6 (2.2)	0.858	
Quality of Life	7.4 (1.5)	6.2 (2.4)	0.006 **^a^	7.4 (1.6)	6.6 (1.9)	0.073	7.3 (1.7)	7.1 (1.8)	0.715	
Happiness	7.2 (1.8)	6.2 (2.4)	0.079	7.4 (1.8)	6.2 (2.0)	0.008 **^a^	7.3 (1.7)	7.0 (2.0)	0.541	
Cancer Treatment Distress	6.8 (2.4)	7.7 (1.2)	0.224	7.1 (2.3)	6.8 (2.1)	0.61	7.0 (2.4)	7.0 (2.2)	0.972	
Satisfaction	7.0 (1.9)	6.2 (2.4)	0.195	7.2 (1.7)	6.0 (2.2)	0.013 *^a^	7.2 (1.7)	6.6 (2.0)	0.204	
Hopefulness	7.1 (1.9)	4.6 (2.6)	0.000 **^a^	7.0 (2.1)	6.5 (2.3)	0.32	6.7 (2.3)	6.9 (2.1)	0.715	
Overall Physical Health	6.9 (1.3)	4.8 (1.9)	0.000 **^a^	6.8 (1.4)	6.0 (2.0)	0.049 *	6.7 (1.5)	6.5 (1.6)	0.603	
Interference Activities at Home	6.4 (2.8)	7.0 (2.5)	0.448	6.4 (2.8)	6.5 (2.5)	0.856	6.3 (2.6)	6.5 (2.8)	0.784	
**(c)**										
	**Disease Phase**			**Age**						
	**Active Treatment**	**Completed Treatment**	***p*-Value**	**Emerging Adults**	**Young Adults**	***p*-Value**				
Support from Others	8.0 (1.7)	8.1 (1.8)	0.77	8.3 (2.0)	8.0 (1.6)	0.43				
Distress Initial Cancer Diagnosis	7.6 (2.7)	7.5 (2.4)	0.88	6.9 (2.5)	7.9 (2.4)	0.06				
Distress for Family	7.2 (2.8)	7.8 (1.6)	0.26	7.7 (1.8)	7.6 (2.1)	0.83				
Quality of Life	6.6 (1.9)	7.4 (1.6)	0.05	7.5 (1.5)	7.0 (1.8)	0.22				
Happiness	6.7 (1.8)	7.2 (1.9)	0.34	7.4 (1.7)	6.9 (2.0)	0.27				
Cancer Treatment Distress	6.5 (2.7)	7.1 (2.2)	0.33	6.9 (2.5)	6.9 (2.2)	0.89				
Satisfaction	6.6 (2.0)	6.9 (1.9)	0.48	7.2 (1.8)	6.7 (2.0)	0.27				
Hopefulness	6.4 (2.0)	6.9 (2.3)	0.32	7.1 (2.1)	6.6 (2.3)	0.36				
Overall Physical Health	6.3 (1.5)	6.7 (1.6)	0.29	6.8 (1.4)	6.4 (1.6)	0.34				
Interference Activities at Home	6.3 (2.1)	6.5 (2.9)	0.8	6.6 (2.9)	6.3 (2.6)	0.64				

(**a**) HRQoL: health-related quality of life; SD: standard deviation; * *p* < 0.05; ** *p* < 0.01; ^a^ = clinically relevant; ^b^ = testicular vs. sarcoma; ^c^ = testicular vs. gynaecologic; ^d^ = testicular vs. other; ^e^ = gynaecologic vs. sarcoma; ^f^ = gynaecologic vs. breast; ^g^ = gynaecologic vs. hematologic; ^h^ = sarcoma vs. other; ^i^ = breast vs. other; ^j^ = hematologic vs. other; ^k^ = sarcoma vs. breast; ^l^ = sarcoma vs. hematologic; ^m^ = testicular vs. breast; ^n^ = testicular vs. hematologic. (**b**) HRQoL: health-related quality of life; SD: standard deviation; * *p* < 0.05; ** *p* < 0.01; ^a^ = clinically relevant; (**c**) HRQoL: health-related quality of life; SD: standard deviation.

## Data Availability

Data available on request due to restrictions (privacy or ethical).
